# The Diet Quality Questionnaire (DQQ) Is a Straightforward Data Collection Tool for Assessing Minimum Dietary Diversity for Women (MDD-W)

**DOI:** 10.1016/j.cdnut.2025.107451

**Published:** 2025-04-25

**Authors:** Olutayo Adeyemi, Muhammad Momoh, Jonathan Makka, Richard Oladipo

**Affiliations:** 1 Department of Human Nutrition and Dietetics, University of Ibadan, Ibadan, Nigeria; 2 Advancing Local Dairy Development in Nigeria (ALDDN) Programme, Sahel Consulting Agriculture and Nutrition Limited, Abuja, Nigeria

**Keywords:** dietary assessments, food systems, dietary diversity, diet quality, DQQ, MDD-W, Africa

## Abstract

Diet quality is an important cause as well as outcome of food systems and global development challenges. There is an increasing demand for diet quality indicators that can be readily assessed. Traditional methods of assessing diets based on quantitative 24-h dietary recall are time consuming, costly, and require specialized skills. Dietary recall approaches that collect information about the consumption of food groups are growing in popularity as they can be used to compute timely indicators of diet quality at much lower costs. Validated indicators that use this method are also becoming more available. The percentage of women of reproductive age who achieve minimum dietary diversity (MDD-W) of ≥5 of 10 predefined food groups is one such indicator and is a proxy for higher micronutrient adequacy of diets. MDD-W has been adopted as an indicator of the sustainable development goals (SDGs). Valid, feasible, and comparable approaches for collecting data for MDD-W calculation across low-, middle-, and high-income countries are of paramount importance in tracking changes in the indicator. The diet quality questionnaire (DQQ) that uses a sentinel foods, close-ended list-based method offers a reliable approach for collecting and analyzing data for MDD-W. Dairy Development Programs implemented in Nigeria by Sahel Consulting Agriculture and Nutrition Limited have used different methods to collect MDD-W data between September 2017 and December 2024. Methods used have included quantitative 24-h dietary recall, nonquantitative open-recall, open-ended lists, closed-ended extensive lists, and DQQ. Data collection and analysis using DQQ have provided the most cost-effective and time-saving approach to obtaining valid results. DQQ can increase the ability of countries and programs to monitor MDD-W and other diet quality indicators. Low- and middle-income countries like Nigeria may consider integrating the DQQ in national data systems to facilitate reporting on MDD-W for progress tracking toward the SDGs.

## Introduction

Measuring diet quality remains an ongoing concern for efforts to improve nutrition globally [1]. Understanding factors associated with malnutrition, identifying populations at risk of malnutrition, designing nutrition education programs and other strategies to improve nutrition, and monitoring/evaluating outcomes of food system interventions, all require the ability to measure diet quality with some level of accuracy [2,3]. Tracking population-level vulnerability, burden of unhealthy diets, changes over time, at-risk groups, intervention needs, and effectiveness of interventions, as well as data-driven advocacy can be achieved even with imperfect dietary indicators [1,4,5]. Yet, tools and indicators for measuring diet quality remain limited and affected by various challenges, such as cumbersome data collection, prohibitive costs, lack of necessary facilities/infrastructure, lengthy processing and data analysis, and inadequate standardization of approaches, tools, and indicators [[6], [7], [8]]. Increasingly, these challenges are being addressed, including through the innovative development of indicators and attendant tools suited to resource-constrained environments, standardization of existing methods, harnessing of advancements in telecommunications and digital tools, and complementarily combining methods [[7], [8], [9]].

One relevant indicator is the percentage of women of reproductive age in a population who achieve the minimum dietary diversity for women (MDD-W) by consuming ≥ 5 of 10 defined food groups. The percent who achieve MDD-W has been validated to be a proxy for the micronutrient adequacy of women’s diets [[10], [11], [12]]. MDD-W is based on whether or not specific food groups were consumed in the previous day and night. The indicator was designed for population-level assessments of whether diets are likely to be adequate in micronutrients. Factors that have been associated with achieving MDD-W include household food security [[13], [14], [15], [16]], nutrition knowledge [17], household income [18,19], agricultural production diversity [13,14], and agricultural season [20,21]. MDD-W is, therefore, a valuable indicator for understanding women’s nutrition, its determinants, and opportunities to improve nutrition. MDD-W is1of 4 indicators recommended for national and global monitoring of healthy diets for purposes including assessing the burden of unhealthy diets, monitoring changes in diets, early warning of disruptions in diets, identification of at-risk groups for targeting, determining causes and consequences of poor diets, designing interventions, and evaluating the effectiveness of interventions [1,5]. MDD-W has recently been approved as an indicator of the Sustainable Development Goals (SDGs) for measuring progress in nutrition [22].

Nevertheless, some authors have highlighted that data collection for MDD-W calculation is prone to inaccurate estimations of diet quality, as a result of potential overreporting for some food groups, underreporting of others, and/or misclassification of foods into food groups [4,[23], [24], [25], [26], [27]]. There is thus a need to identify ways to improve data collection to facilitate higher data accuracy, while maintaining simplicity and ease of data collection. The perspective presented in this paper highlights the value of using a recently developed tool—the diet quality questionnaire (DQQ) [28] to collect data for MDD-W calculation. The perspective describes the MDD-W and DQQ, and reports experiences from a case study which found that the DQQ enabled MDD-W data collection more efficiently and effectively than other methods tried.

## Established Methods for Collecting MDD-W Data

There are 2 primary ways through which MDD-W data can be collected, open-recall or list-based methods [12,29]. Both methods require preparatory work before data collection, to produce a local list of foods categorized according to the MDD-W food groups. The food list for the open-recall is extensive, including nearly all possible options for foods in each food group, whereas the list-based method uses a shorter list of the most frequently consumed foods [12]. When implemented correctly, both open-recall and list-based methods generate comparable results [24,25,30].

The open-recall method requires respondents to report all foods and beverages consumed the previous day and night. The enumerator records these foods and selects the corresponding food group on a predefined list [12,29]. With high-quality implementation, this method produces complete recall and allows probing to ensure that the amounts of various foods consumed is ≥ 15 g, as recommended to avoid overcounting foods that do not contribute significantly to nutrient intakes [12,29]. However, the open-recall requires skilled enumerators who can accurately classify foods and greater enumerator training. The open-recall is also more challenging to use with electronic data collection and can be cumbersome in large surveys [12,29].

With the list-based method, a list of foods per food group is read to the respondent by the enumerator, and the respondent responds “yes” when they hear foods/food groups that they have consumed the preceding day and night. The list-based method is easier to collect electronically and in large surveys and requires less enumerator training. MDD-W can however be overestimated with the list-based method because respondents may report foods consumed in very small amounts (<15 g) that should not count [4,25].

List-based methods can use open-ended food list questions or close-ended questions [27]. Open-ended lists, such as included in initial MDD-W guidelines [29], phrase food lists in the manner of this example—“foods made from grains like wheat, maize, sorghum, rice, or other foods made from grains.” Such lists place a high cognitive burden on respondents to correctly identify food groups corresponding to foods consumed, and are susceptible to misclassification of foods [4,27,31,32]. Close-ended lists (for example, “orange, tangerine, or grapefruit”) have less cognitive burden [27] but may exclude some foods [4]. Current MDD-W guidelines recommend adopting close-ended lists as much as possible, and identifying and using sentinel foods [12].

## DQQ Tool for Collecting MDD-W Data

DQQ data collection uses the closed-ended, sentinel foods list-based method. Sentinel foods are context- and food group-specific foods that account for a high percentage of people who have consumed the food group [33,34]. Existing work has already adapted (and translated into national languages) the DQQ tool for >140 countries [33]. The DQQ collects data about the consumption of 29 food groups, can be administered in 5 min, was adapted for use among adult men and women, and can be used to calculate a suite of indicators that address both nutrient adequacy as well as dietary risks of noncommunicable diseases (NCDs) [28]. MDD-W is one of the indicators that can be calculated using the DQQ.

The 29 food groups in the DQQ include snacks, deep-fried foods, processed meat and other food groups that are associated with risk of NCDs. Food groups that are protective against NCDs are also included, such as whole grains and several fruit and vegetable subgroups. These food groups facilitate the calculation of NCD-Risk and NCD-Protect scores that have been validated based on WHO recommendations [28,35,36]. NCD-Risk and NCD-Protect scores are used together in computing the Global Dietary Recommendation score, another of 4 indicators recommended for national and global monitoring of healthy diets [1,5]. The consumption of all 5 food groups commonly recommended for daily consumption in food-based dietary guidelines is another indicator that can be assessed using DQQ [28,35]. Thus, in addition to the benefits of closed-ended list-based methods highlighted earlier, using the DQQ for MDD-W data collection is cost-effective because additional indicators to better understand diet quality can simultaneously be covered [28]. The DQQ tool has now been used for MDD-W data collection in 94 countries [33].

Although data from open-recall methods could potentially be categorized into the DQQ groups to calculate the additional indicators, evidence shows that snacks and unhealthy items are often poorly recalled in open-recall [4], creating challenges for calculating NCD-Risk scores. Moreover, further adaptation would need to be done to open-recall methods to calculate all DQQ indicators, including probing food preparation and food sources so that deep-fried foods and packaged salty snacks can be identified. The time involved in having to categorize open recalls into 29 food groups would likewise be considerable.

Available studies comparing how the DQQ performs against a multipass quantitative 24-h dietary recall found that differences were small and generally not significant between the DQQ and the quantitative 24-h recall in the percent of women who achieved MDD-W [37]. Still, the DQQ has been adapted at the country level and may exclude sentinel foods consumed by subpopulations within a country, for example, some ethnic groups. It is therefore important for the DQQ food list to be examined in the local context, and further adapted if needed, when used among subpopulations within a country [4,33]. Like all dietary assessment methods that rely on respondents’ report, the DQQ can be affected by recall and social desirability bias. Such bias can be minimized by training enumerators to adhere to data collection guidelines, establish rapport with respondents, remain neutral in verbal and nonverbal reactions, and collect data as privately as possible [4,12,31].

The DQQ addresses several limitations of open-recall and open-ended list-based methods [28,33,37]. The tool does not require any further adaptation when used at country level (for the 140 countries with an adapted tool), thereby reducing preparatory work before data collection and saving time and costs [33]. DQQ is easy to program electronically and does not require enumerators who are skilled in categorizing foods into food groups. Unlike the open-ended list, there is no cognitive burden for respondents to categorize foods into food groups [28,33]. With the DQQ, ≤7 sentinel foods per question are read to respondents, avoiding challenges of respondents forgetting some mentioned foods or becoming fatigued when close-ended food lists are extensive [33]. Challenges with overestimation of MDD-W as a result of respondents reporting foods typically consumed in amounts <15 g (for example, condiments and ingredients for flavor) are also minimized with the DQQ because foods consumed in such small amounts are not included in DQQ lists [28,33,37]. Where sentinel foods are idiosyncratically consumed by a large percentage of people in amounts <15 g, the DQQ may overestimate the consumption of the affected food group [37], but this is a limitation of nonquantitative list-based methods in general.

## Case Study of MDD-W Data Collection for Project Monitoring and Evaluation in Nigeria

Sahel Consulting Agriculture and Nutrition Limited implemented the 2017–2019 Nigerian Dairy Development Program (NDDP), including a 2017 assessment of nutrition status and determinants for women and children in program communities. The assessment aimed to identify entry points to improve the nutrition of smallholder dairy producers [38] and MDD-W was one of the indicators assessed. Experiences from the NDDP were used to design the 2019–2025 Advancing Local Dairy Development in Nigeria (ALDDN) program, which includes objectives to improve dietary diversity of 60,000 people living in smallholder dairy households and 60,000 people from nondairy households. Dairy households receive livelihoods and home gardening support and nutrition education. Nondairy households primarily receive just nutrition education [38]. MDD-W is a key outcome indicator for ALDDN. ALDDN staggered the enrollment of participating households. Process and output monitoring was therefore conducted at different times for cohorts of households enrolled in the program. Between January 2021 and December 2024, survey data were collected 6 times as part of monitoring and evaluation activities for ALDDN. Methods used to collect MDD-W varied each round of data collection, until December 2022 when the DQQ was first and subsequently used. Details about the experiences collecting data for MDD-W calculation are in Table 1, highlighting approaches, results, and challenges.

**TABLE 1:** Overview of MDD-W use in the Nigerian Dairy Development Program (NDDP) and Advancing Local Dairy Development in Nigeria (ALDDN) Program in surveys between September 2017 and December 2024.

Project and purpose	Date	Survey team and training	MDD-W sample and sample size	MDD-W data collection method	MDD-W results	Remarks
NDDP assessment of nutrition status and determinants. Multitopic survey.	September–November 2017	External national firm that recruited enumerators with prior nutrition data collection training and trained them for 2 d. Trainers included 2 nutritionists skilled in collecting dietary data	503 women from smallholder dairy households in Oyo and Kano States	Close-ended extensive list-based method adapted from 2016 MDD-W guidelines [29]. Paper data collection.	Score: 9.6 ± 1.0 % achieved MDD-W: 99.6%	Two methods were used to collect dietary data in the same survey, to assess MDD-W and nutrient intakes. The MDD-W score from data collected using close-ended extensive foods list was implausibly high. Enumerators reflected that the women invariably responded “yes” to the consumption of a food group when a long list of foods was read to them. Quantitative 24-h recall was thus coded into the MDD-W food groups during analysis and used for MDD-W calculation.
			490 of the 503 women from smallholder dairy households in Oyo and Kano States	Quantitative multipass 24-h dietary recall. Paper data collection.	Score: 5.1 ± 1.2 % achieved MDD-W: 66.3%	
ALDDN evaluation baseline assessment. Multitopic survey.	January–March 2021	Independent international research and evaluation firm working with a global data collection firm with Nigeria experience. Enumerators trained for 4 d, including pilot of survey and feedback.	2453 women from smallholder dairy households in Adamawa, Kaduna, Kano, and Plateau States	Open-ended list-based method. Electronic data collection.	Score: 5.8 ± 2.0 % achieved MDD-W: 71.5%	Food list was similar to the MDD-W list in the 2018 Nigeria Demographic and Health Survey [40], which had been based on country list adaptations done by the FAO of the United Nations in 2015 [41]. Tool worked well for calculating MDD-W, but it is not possible to tell whether misclassification by respondents occurred [4,27,31]. The training of enumerators (4 d) was also longer than is feasible for routine monitoring and evaluation by the project.

ALDDN process and outputs monitoring. Nutrition and hygiene knowledge, attitude, and practice survey.	September–November 2021	ALDDN monitoring and evaluation team working with community-level enumerators with tertiary education who were trained for 1 d.	1735 women from smallholder dairy households in Adamawa, Kaduna, Kano, and Plateau States	Nonquantitative open-recall method Electronic data collection.	Unable to calculate	Coding into MDD-W food groups was to be done during data analysis. In many instances, enumerators did not list ingredients in mixed dishes, or did not probe items like pepper to determine whether respondent consumed red bell peppers (vitamin A-rich vegetable), habanero peppers (other vegetables), or both, and both vegetables are ubiquitously consumed in meaningful quantities in Nigeria. It was therefore impossible to code the data into the MDD-W food groups.

ALDDN evaluation midline assessment. Multitopic survey.	March–May 2022	Independent international research and evaluation firm working with a global data collection firm with Nigeria experience. Enumerators trained for 4 d, including pilot of survey and feedback.	1841 women from smallholder dairy households in Kaduna, Kano, and Plateau States	Open-ended list-based method. Electronic data collection.	Score: 5.6 % achieved MDD-W: 69.0%	The same approach as the baseline evaluation assessment was used.

ALDDN process and outputs monitoring. Program households’ census and diet quality survey.	December 2022	ALDDN monitoring and evaluation team working with university students as enumerators who were trained for 1 d.	6436 women from smallholder dairy households in Adamawa, Jigawa, Kaduna, Kano, and Plateau States	DQQ method, using publicly available Nigeria-adapted DQQ tool obtained from the DQQ website [39]. Electronic data collection.	Score: 5.9 ± 2.2 % achieved MDD-W: 71.1%	The DQQ list was reviewed by enumerators who were indigenes of the survey state to which they were assigned, to ensure that sentinel food lists were complete and translations into local language were accurate. Sample was selected from ALDDN households that were enrolled from 2020 to 2022.

ALDDN process and output monitoring. Nutrition, water, sanitation, hygiene, and food safety knowledge, attitude, and practice survey.	September 2023	ALDDN monitoring and evaluation team working with university students as enumerators who were trained for 1 d.	650 women from smallholder dairy households in Adamawa, Jigawa, Kaduna, Kano, and Taraba States	DQQ tool. Electronic data collection.	Score: 6.7 ± 2.3 % achieved MDD-W: 80.3%	Sample selected from ALDDN participating households that were enrolled in 2023.

ALDDN process and output monitoring. Diet quality survey.	February 2024	ALDDN monitoring and evaluation team working with university students as enumerators who were trained for 1 d.	1457 women from smallholder dairy households in Adamawa, Jigawa, Kaduna, Kano, and Plateau States	DQQ tool. Electronic data collection.	Score: 5.8 ± 2.0 % achieved MDD-W: 70.6%	Sample was a subset of the December 2022 sample.

Abbreviations: DQQ, diet quality questionnaire; MDD-W, minimum dietary diversity for women.

## Lessons Learned from Experiences Collecting MDD-W Data

DQQ provided a straightforward, efficient, and practical approach to collecting MDD-W data. First, the availability of standardized, sentinel, and translated food lists for Nigeria meant that the tool required minimal effort to ensure that no sentinel foods in the project communities had been omitted. (English food lists and translations into the 3 major languages in Nigeria are available on the Global Diet Quality Project website [39]). Additionally, the avoidance of long food lists meant that overestimation of MDD-W, as was experienced using the extensive list-based method in the NDDP study (Table 1), was minimized. Second, training to use the DQQ was much more straightforward for the M&E team than training for the open-recall or open-ended list-based MDD-W methods, and there were fewer ambiguities and confusion for enumerators without dietary data training. Third, DQQ data took less time to collect than other methods given survey length, sample size, and number of enumerators. Fourth, respondents did not have any trouble understanding and responding to the DQQ questions — with open-recall approaches, respondents asked more clarifying questions. Fifth, the availability of an online indicator calculator on the Global Diet Quality Project website [39] meant that the M&E team could process the DQQ data quickly and autonomously. Moreover, the lower training requirements and data collection/analysis time savings afforded by DQQ meant that the cost implications of surveys were lower. The advantage of other indicators in addition to the MDD-W was also important because it provided a more holistic view of diets than the MDD-W alone could give. Finally, the use of the same tool over time ensured consistency, minimizing bias that could arise from differences in data collection methods, and facilitated temporal comparisons.

The high financial, time, and capacity costs of weighed food records or full quantitative, rigorous, multiple-pass 24-h recalls, which are reference standard dietary intake assessment methods [25,37], were prohibitive in the context of routine monitoring of this non-research, nutrition-sensitive food systems intervention, and such a level of accuracy was also unnecessary in this context. Though nonquantitative open-recall methods required fewer resources, our experience showed that the capacity requirement for adequate implementation of this method could still be a critical limiting factor. The DQQ tool is a lower-cost, time-saving tool that does not require additional skills beyond those required for survey best practices, and has been found to be invaluable for understanding and monitoring diet quality, including MDD-W. The tool could be particularly useful for nutrition-sensitive programs and interventions that need to routinely assess diet quality indicators, including indicators of both healthy and unhealthy diets, and for men and not just women.

## Conclusion

Food group–based dietary intake assessment indicators such as the MDD-W are gaining increasing importance because of their simplicity and feasibility in regular tracking of diet quality compared with more precise but time-consuming and costly approaches. MDD-W data can be collected using open-ended list-based, close-ended extensive list-based, or open-recall methods, but a program in Nigeria found that these methods can be problematic in ensuring accurate results when enumerators do not have nutrition and dietary assessment training and experience. The DQQ tool, which uses sentinel foods, closed-ended list-based method in line with current guidelines for collecting MDD-W data was an alternative that was easy to train for, administer, and analyze. Although recall and social desirability biases are potential challenges for assessing diet quality using DQQ, these challenges can be minimized by use of neutral wording and neutral verbal and nonverbal reactions to responses.

Low- and middle-income countries like Nigeria may consider integrating the DQQ in national data systems to facilitate calculation of MDD-W and other diet quality indicators for assessing the burden of unhealthy diets, monitoring changes in diets, identifying at-risk groups, and evaluating the effectiveness of interventions, as well as progress tracking for the SDGs. Food systems-related and nutrition education interventions in these countries may also consider including the DQQ tool in routine M&E systems as a low-burden option to enhance understanding of project outcomes related to healthy and unhealthy diets among various population groups and gaps that could be addressed through program design and implementation.

## Author contributions

The authors’ responsibilities were as follows – OA: led the writing and revision of the manuscript; and all authors: were responsible for the conceptualization, development, and final content of the manuscript, read and approved the final manuscript.

## Data availability

NDDP and ALDDN data referenced in the article will be made available on request.

## Funding

This work, and the NDDP and ALDDN programs described therein, was supported, in whole, by the Gates Foundation (grant number OPP1215286). The conclusions and opinions expressed in this work are those of the authors alone and shall not be attributed to the Foundation. Under the grant conditions of the Foundation, a Creative Commons Attribution 4.0 License has already been assigned to the Author Accepted Manuscript version that might arise from this submission.

## Conflict of interest

OA reports a relationship with the Global Alliance for Improved Nutrition that includes consulting or advisory related to integration of the diet quality questionnaire in Nigeria data systems. OA worked with the FAO of the United Nations from 2015 to 2019 and was involved in inception activities to institutionalize the minimum dietary diversity indicator in Nigeria. If there are other authors, they declare that they have no known competing financial interests or personal relationships that could have appeared to influence the work reported in this paper.
